# The Origin of Mathematics and Number Sense in the Cerebellum: with Implications for Finger Counting and Dyscalculia

**DOI:** 10.1186/s40673-017-0070-x

**Published:** 2017-07-20

**Authors:** Larry Vandervert

**Affiliations:** American Nonlinear Systems, Spokane, WA USA

**Keywords:** Cerebellar inverse dynamics models, Cerebellar somatotopic maps, Cerebellum, Developmental dyscalculia, Finger counting, Inner speech, Mathematics, Number sense, Verbal working memory

## Abstract

**Background:**

Mathematicians and scientists have struggled to adequately describe the *ultimate foundations* of mathematics. Nobel laureates Albert Einstein and Eugene Wigner were perplexed by this issue, with Wigner concluding that the workability of mathematics in the real world is a mystery we cannot explain. In response to this classic enigma, the major purpose of this article is to provide a theoretical model of the ultimate origin of mathematics and “number sense” (as defined by S. Dehaene) that is proposed to involve the learning of inverse dynamics models through the collaboration of the cerebellum and the cerebral cortex (but prominently cerebellum-driven). This model is based upon (1) the modern definition of mathematics as the “science of patterns,” (2) cerebellar sequence (pattern) detection, and (3) findings that the manipulation of numbers is automated in the cerebellum. This cerebro-cerebellar approach does *not* necessarily conflict with mathematics or number sense models that focus on brain functions associated with especially the intraparietal sulcus region of the cerebral cortex. A direct corollary purpose of this article is to offer a cerebellar inner speech explanation for difficulty in developing “number sense” in developmental dyscalculia.

**Results:**

It is argued that during infancy the cerebellum learns (1) a first tier of internal models for a primitive physics that constitutes the foundations of visual-spatial working memory, and (2) a second (and more abstract) tier of internal models based on (1) that learns “number” and relationships among dimensions across the primitive physics of the first tier. Within this context it is further argued that difficulty in the early development of the second tier of abstraction (and “number sense”) is based on the more demanding attentional requirements imposed on cerebellar inner speech executive control during the learning of cerebellar inverse dynamics models. Finally, it is argued that finger counting improves (does not originate) “number sense” by extending focus of attention in executive control of *silent* cerebellar inner speech.

**Discussion:**

It is suggested that (1) the origin of mathematics has historically been an enigma only because it is learned below the level of conscious awareness in cerebellar internal models, (2) understandings of the development of “number sense” and developmental dyscalculia can be advanced by first understanding the ultimate foundations of number and mathematics do not simply originate in the cerebral cortex, but rather in cerebro-cerebellar collaboration (predominately driven by the cerebellum).

**Conclusion:**

It is concluded that difficulty with “number sense” results from the extended demands on executive control in learning inverse dynamics models associated with cerebellar inner speech related to the second tier of abstraction (numbers) of the infant’s primitive physics.

## Background

Historically, scientists and mathematicians have been mystified by the workability of mathematics in the “real world.” Einstein [1] asked, “How can it be that mathematics, being after all the product of human *thought* [italics added] which is independent of experience, is so admirably appropriate to the objects of reality?” This same question, though stated quite differently, is explicit in this title of a classic article by another Noble laureate, Eugene Wigner [2], “The Unreasonable Effectiveness of Mathematics in the Natural Sciences.” Wigner concluded his article with this statement of puzzlement:

The miracle of the appropriateness of the language of mathematics for the formulation of the laws of physics is a wonderful gift which we neither understand nor deserve. We should be grateful for it and hope that it will remain valid in future research and that it will extend, for better or for worse, to our pleasure even though perhaps also to our bafflement, to wide branches of learning. (p. 14)

More recently, leading computer scientist and mathematician, Derek Abbott [3], proposed that the origin of mathematics is not a mystery, but rather is a product of the human mind. Abbott argued strongly against what he calls “mathematical Platonism,” the idea that mathematics has a mysterious, independent existence from the human mind. However, as an alternative he was only able to conclude that, “Mathematics is a product of the imagination that sometimes works on simplified models of [the regularities found in] reality,” ([3], p 2152). He did not specify how in imagination these models of mathematics might originate or how we might come to know of them.

## Purpose

The purpose of this article is to describe how newer understandings of the prominent role of the human cerebellum in the cerebro-cerebellar development of science (Vandervert, [4]) and in culture (Vandervert, [5]) can be extended to provide a way to address the long-standing “mystery” of the workability of mathematics in the real world. Mathematics has recently developed beyond its historical definitions of number and geometry to be understood as “the science of patterns:”

Mathematics is the science of patterns. The mathematician seeks patterns in number, in space, in science, in computers, and in imagination. Mathematical theories explain the relations among patterns; functions and maps, operators and morphisms bind one type of pattern to another to yield lasting mathematical structures. Applications of mathematics use these patterns to “explain” and predict natural phenomena that fit the patterns. ([6], p. 616)

See also Devlin’s [7] description of mathematics as the science of patterns which includes the study of the patterns of shape, motion, number, and behavior.

In recent decades abundant imaging research has found that the cerebellum is a master computational system for both motor and cognitive areas of the cerebral cortex [8, 9]. Within the context of these findings and within the context of mathematics as the science of patterns, it is proposed that the cerebellum (1) computes sequences of *patterns* that predict future states of affairs in movement and thought, (2) does this by learning *internal models* of the *patterns* in the physical and internal worlds which first produce primitive physics in the infant [4, 5], and (3) in a second tier of distillation of this primitive physics (and all subsequently derived physics), learns internal models behind the origin and manipulation of the *patterns* of mathematics (shape, motion, behavior and “number”). Specifically, this article lays out newer cognitive neuroscience findings on the functions of the human cerebellum that describe the computational mechanisms of silent (below conscious awareness) *inner speech* within working memory in the cerebro-cerebellar system. These computational mechanisms will be used to directly support Abbott’s [3] view that mathematics is not a mystery but is a product of simplified models of reality in human “imagination” (in both movement and thought). In collaboration with the cerebral cortex, mathematics, the science of patterns, is the product of (1) the cerebellum’s fundamental sequence (or pattern) detection of internal and external events, and (2) the cerebellum’s optimization (through constantly error-corrected patterning) of prediction [8, 9].

A corollary to the foregoing larger purpose of this article is to offer an explanation for difficulty in developing what Stanislas Dehaene [10] called *number sense,* and the cerebro-cerebellar origins of developmental *dyscalculia*. Dehaene used the term number sense “as a shorthand for our ability to quickly understand, approximate, and manipulate numerical quantities” ([10] (p. 16)), and number sense will be limited to that meaning for the purposes of this article. Dyscalculia refers to difficulties in learning or comprehending arithmetic—more will be said of dyscalculia later in this article. It will be proposed that difficulties in acquiring number sense and the difficulties in dyscalculia are the result of developmental problems associated with the learning of cerebellar *inverse dynamics models* (which operate below the level of conscious awareness) associated with the second tier of the primitive physics of the infant (of number) mentioned in the preceding paragraph*.*

Dehaene [10] made a strong point of the speedy “intuitive” nature of number sense: “I collectively refer to those fundamental elementary [numerical] abilities or intuitions as number sense,” (p. 616). He attributes this quick, intuitive character of number sense to specifically evolved brain regions of the cerebral cortex. However, it will be shown how these elementary abilities or intuitions for number might be more parsimoniously and more definitively be explained by the above-described cerebellum-driven mechanisms of unconscious learning.

### A note on the traditional attribution of number sense to the Intraparietal Sulcus region of the cerebral cortex

It is important to note before moving on to a discussion of the broader background of cerebro-cerebellar coordination that the cerebro-cerebellar approach does *not* necessarily conflict with mathematics or number sense models that focus on brain functions associated with especially the intraparietal sulcus region of the cerebral cortex, for example, Dehaene [10], Dehaene, Spelke, Stanescu, Pinel and Tsivkin [11]. Rather, the cerebro-cerebellar approach brings to bear additional brain mechanisms that provide more detailed and more comprehensive explanations for (1) the initial learning of number and its manipulation, and (2) the subsequent, ongoing optimization and increased complexity of the neural patterns that constitute both mathematics and number sense. It will be seen in the next section that the cerebro-cerebellar approach provides these same advantages in all areas of the movement and thought in the cerebral cortex.

For these reasons a discussion of these strictly cerebral cortex-based models would be beyond the scope and space limitations of this article. We will return to this point in regard to developmental dyscalculia later in the article.

### The recent evolution of Cerebro-Cerebellar coordination toward the learning and optimization of mental processes

To help understand the arguments of this article on how the brain creates mathematics and number sense, it will be helpful to review research on cerebro-cerebellar collaboration toward the learning and optimization of mental processes. In their watershed articles, Leiner, Leiner and Dow [12, 13] began by noting that the human cerebellum increased three- to fourfold in last million or so years. They further pointed out that this huge increase in size of the cerebellum included the further evolutionary development of two-way nerve tracks (20 million on each side of the brain) linked to the cerebral cortex, including to the parietal and prefrontal areas for planning and language functions (Leiner, Leiner & Dow [13]). Within this cerebro-cerebellar framework, Leiner, Leiner and Dow proposed that the evolutionarily differentiated development of the newer part of the cerebellum’s dentate nucleus (the *ventral* dentate) enabled the brain to unconsciously manipulate *ideas and their communication* with great dexterity just as the phylogenetically older portion of the dentate nucleus (the *dorsal* dentate) had done for motor skills. Today, such unconscious manipulation of ideas in the newer ventral dentate has been referred to as unconscious internal speech processes that enhance verbal working memory (Gilchrist, [14]; Marvel & Desmond, [15–17]).

Leiner, Leiner and Dow’s [12.13] foregoing early speculations and hypothesis concerning the cognitive functions of the cerebellum have been strongly supported by literally hundreds of brain-imaging and clinical studies. Among such studies particularly relevant to the present article are the following: Akshoomoff, Courchesne and Townsend [8]; Balsters, Whalen, Robertson et al. [18]; Ito [19–21]; Leggio and Molinari [9]; Liao, Kronemer, Yau et al. [22]; Marvel and Desmond [15, 16]: Schmahmann [23]; Stoodley, Valera and Schmahmann [24]; Strick, Dum and Fiez [25]; van Dun, Manto and Mariën [26]. Van Dun, Manto and Mariën provide particularly salient background on the error-corrective prediction via language functions of the cerebellum.

**Fig. 1:**
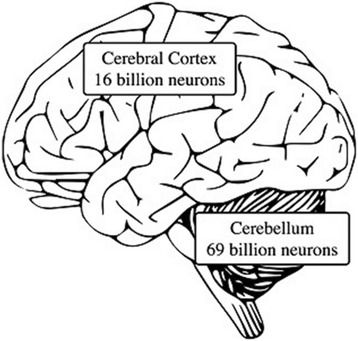
Illustration of the cerebellum in relation to the cerebral cortex along with their respective neuron counts. The neuron counts are based on Lent R, Azevedo FAC, Andrade-Moraes CH, Pinto AVO [27]

Figure 1 illustrates the enormous, 69-billion-neuron computational capacity of the cerebellum compared to 16 billion neurons in the cerebral cortex [27] that, through generation-after-generation of repetitious (or practiced) behavior and thought and thereby constantly advancing skills and mental models, is proposed to have been and continues to be behind the evolution of uniquely human culture, including language and mathematics.

In humans, the ventral dentate is twice as large as the dorsal dentate and is proportionately larger than that of the great apes (Bostan, Dum & Strick [28]). Marvel and Desmond [15] suggested that the newer ventral dentate (cognitive loop) was naturally selected *from* the evolutionarily older dorsal dentate (motor loop) as the cerebellar cortex and frontal areas of cerebral cortex expanded over the last million years. The ventral dentate of the cerebellum outputs to the frontal and parietal areas of the cerebral cortex (working memory, executive functions including planning, and rule-based learning).

Via the dentate nucleus, then, the cerebellum is involved in the learning of countless internal models which are sent to the cerebral cortex for both motor and cognitive processing. Based on extensive research studies, Bostan, Dum and Strick [28] argued that the “signal from the dentate to the prefrontal and posterior parietal areas of the cortex [working memory, executive functions and rule-based learning] is as important to their function as the signal the nucleus sends to motor areas of the cerebral cortex” (p. 3). Thus, as a 69-billion-neuron-strong computational system based on sequence detection and prediction (Leggio & Molinari [9]), the human cerebellum wields an “unconscious presence” in thought, behavior and affect. As Bostan, Dum and Strick intimated above, the cerebellum’s cognitive influence on prefrontal and posterior parietal areas of the cerebral cortex is commensurate with the immense learning requirements and apparently unlimited potential of the initiation and products of culture as proposed by Vandervert [5], and, in this article, of mathematics. We will return to both the cerebellar mechanism of sequence detection and to the yoked ontogenetic cerebellar dorsal-to-ventral dentate development as they apply to all forms of movement, cognition and to the origin of mathematics in some detail in later sections of this article.

### The cerebellum provides a common computational language to movement and cognitive processes (including mathematics) in the cerebral cortex

Through the repetition of movement and cognitive skills (including imaginative thought), the human cerebellum learns progressively more efficient *internal models* of movement and mental processes that are going on in the cerebral cortex (Ito, [19, 20, 29]; Stoodley, Valera & Schmahmann [24], Strick, Dum & Fiez, [25]). These cerebellar internal models consist of *distilled or compressed patterns* of the movement/thought sequences repeatedly taking place in the cerebral cortex. How this “distillation” first takes place in cerebellar models during infancy will be described in in later sections of this article. See Fig. 1 for a recent neuron count and computational capacity of the cerebellum.

During the foregoing distillation process, movement and cognitive skills are reduced in the cerebellum to a common computational language of sequences or patterns that the various specialized areas of the cerebral cortex have evolved to translate toward optimal future behavioral and thought control (Akshoomoff, Courchesne & Townsend, [8]; Leggio & Molinari, [9]; Vandervert, [4, 5, 30]). With repetition, these more efficient cerebellar models, operating below the level of conscious awareness, are to the cerebral cortex to bypass the original arduous, time consuming cerebral cortical circuits; the cerebellar models make all movement/mental skills smoother, quicker, and progressively more error-free (Doya, [31]; Ito, [20, 29]). The cerebellar models are also *blended within and across* skills (Imamizu, Higuchi, Toda & Kawato, [32]; Yomogida, Sugiura, Watanabe et al. [33]) wherever it will make them more efficient and will allow them to *predict* complex movement/mental requirements before they occur (Akshoomoff, Courchesne & Townsend, [8]; Leggio & Molinari, [9]). These efficiency effects of cerebellar internal models on goals formulated in the cerebral cortex are seen in any repeated activity. This includes everything from the progressively more expert skills in playing basketball, musical performance, solving math and engineering problems and so on. This cerebellar prediction process will be described in detail in a moment.

### Breakthrough evidence of the manipulation of numbers (arithmetic) in cognitive areas of the cerebellum

As stated earlier in the purposes section, this article extends the learning of cerebellar internal models to an explanation of (1) the workability of mathematics in the real world, to (2) the manipulation of numbers (arithmetic), and to (3) the origin of number sense (fluidity or automaticity in number use and understanding). Directly in this regard, and following in the vein of the foregoing long line of discovery of the cognitive functions of the cerebellum began by Leiner, Leiner and Dow [12, 13], Hayter, Langdon and Ramnani [34] conducted an imaging study on the involvement of the cerebellum in arithmetic calculation in verbal working memory. Hayter et al. used the Paced Auditory Serial Addition Test (PASAT) where subjects sequentially added the last two numbers in an overall sequence of five numbers. During this number addition task in verbal working memory, it was found that Crus VII in the cerebellar cortex along with prefrontal and parietal areas of the cerebral cortex were involved in *automated* counting. Hayter et al. concluded the following:

We suggest that the cerebellar activation reflects the *automated simulation* [italics added] of cognitive operations [in cerebellar internal models] that are initially reliant on interactions between prefrontal areas, and that interaction between prefrontal areas and their targets is simulated [in internal models] within the circuitry of cerebellar cortical lobule VII.

One salient characteristic of the PASAT is the relatively high demands it places on information [number] management. Numbers must be held in working memory, added to previously heard numbers, and then replaced by the next number. This task clearly requires participants to maintain and manipulate [number] information within working memory. ([34], p. 950)

Hayter, Langdon and Ramnani [34] clearly showed cerebellar cognitive involvement in automated number manipulation in verbal working memory. However, in this very early work on the cerebellum’s role in working memory and number manipulation Hayter et al. did not provide specific analyses of the component phases of working memory (encoding, maintenance, and retrieval) or on inner speech. Later in the dyscalculia section of this article, we will return to more recent studies on working memory in the cerebellum which do include these refinements and which point directly toward the origins of number sense and dyscalculia in verbal working memory inner speech in the cerebellum.

### The ultimate foundations of number and mathematics

#### Physics (beginning in the infant)

In attempting to understand the fundamental nature mathematics, Eugene Wigner, who was quoted at the beginning of this article, and Derek Abbott [3] began by discussing the history of the interlacing of mathematics with physics, the latter (especially its regularities) being our scientific contact point with “reality.” A similar approach will be taken here. Only here it will be done from the point of view of the cerebro-cerebellar loops in the brain and their development of the awaking of the movement and cognitive processes that come to know about physics and mathematics in the first place, namely, the infant’s first and continuing foundational operating system of *working memory*.

Working memory in this article consists of the merging of two theoretical models of working memory. First, is Baddeley’s [35, 36] model which includes (1) a central executive (control of attention), (2) a visual-spatial sketchpad, (3) a phonological or speech loop, and (4) an episodic buffer which links the other components in temporal sequences and connects them with long-term memory. Together, these four components constitute and operational description of current ongoing conscious states during any sort of problem solving, including imagination. Second, to answer the question of how and in what cognitive framework, exactly, problem solving and imagination occur, Cowan [37] argued that working memory is the “cauldron” for *concept formation*, and that, within this cauldron, “the binding of ideas occurs more specifically in the focus of attention” (p. 210). By “binding,” Cowan is referring to the joining of existing concepts together to form new concepts. Within the context of these models of working memory (Baddeley [35, 36] and Cowan [37]) the interrelated phylogenetic and ontogenetic development of working memory is articulated in some detail in Vandervert [4, 38].

### The origin of physics in the brain: The Cerebrocerebellar beginnings of a primitive physics in the working memory in the infant

Vandervert [4, 5, 30, 38] has argued that the most detailed behavioral/cognitive account that can be used to describe how the infant first builds working memory is the considerable research of Mandler [39–42]. She proposed that the infant’s *repetitive* patterns of *noticing* aspects of its own bodily movement in relation to objects moving in the environment (the relationships among objects, space, and time) are “*distilled”* or *“condensed”* [39] into *conceptual primitives*. (By “primitive” Mandler meant foundational, and did not mean unstructured, but structured.) Mandler further proposed two mechanisms that indicate that this noticing and distillation by the infant constituted the beginning of the construction of working memory: (1) noticing/distillation were the result of, “an *attentional mechanism* [italics added] dedicated to simplifying spatiotemporal information” ([42], p. 426), and (2) noticing/distillation form the basis of an *accessible conceptual system* ([40], p. 273). For both Baddeley [35] and Cowan [37], executive *attention* and *access* to conceptual information (via the episodic buffer) are the theoretical earmarks of working memory.

**Fig. 2:**
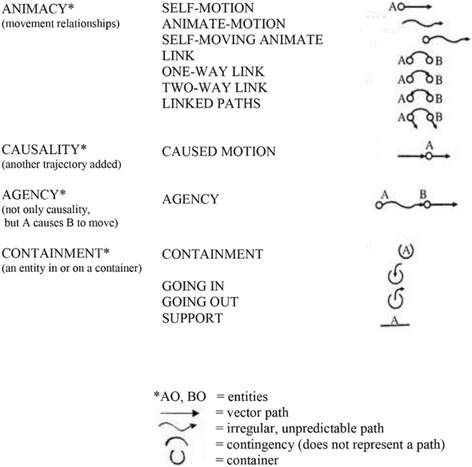
Mandler’s [39–42] conceptual primitives—collectively, the infant’s unconscious “primitive physics”

Figure 2 illustrates Mandler’s characterizations of such “spatiotemporal” conceptual primitives she derived from her extensive experiments with infants. According to Mandler [41], the conceptual primitives shown in Fig. 2 form the foundational basis of potential *consciously accessible visuospatial meanings* for later relational thought and language.

Since, according to Vandervert [4, 43], Mandler’s conceptual primitives (1) are driven into existence by attentional processes in the infant, and (2) are accessible visuospatial meanings, Vandervert [4] argued that, together, they form the initial central executive and *an initial slave componen*t of the infant’s working memory. In other words, Vandervert proposed that, when put in sequential motion by attentional control processes, the conceptual primitives in Fig. 2 provide the infant with a *visual-spatial* working memory*.* Following Mandler’s above theoretical premises, Vandervert [38] proposed that, as the infant develops toward childhood, the visuospatial meanings (Fig. 2) are *blended* in the cerebellum with the infant’s vocalizations in the process of language acquisition.

### Mandler’s conceptual primitives are Cerebellar internal models that can make predictions about future events

Vandervert [4, 30, 44] argued that Mandler’s conceptual primitives are encoded as cerebellar internal models in the infant in accordance with Akshoomoff, Courchesne and Townsend’s [8] and Leggio and Molinari’s [9] cerebellar sequence detection process. Within this framework of the early growth of working memory, it is proposed that cerebellar sequence detection hypothesis proposed by Leggio and Molinari [9] learns the internal models that build the neural patterning of the infant’s initial visual-spatial working memory:

According to this [sequence detection] hypothesis, the cerebellum detects and simulates repetitive patterns of temporally or spatially structured events, regardless of whether they constitute sensory consequences of one’s actions in motor planning, expected sensory stimuli in perceptual prediction, or inferences of higher-order processes (e.g., *cognitive elaboration* [italics added] or social cognition). The simulation allows internal models to be created that can be used to make predictions about future events that involve any component, such as the *body, other persons, and the environment* [italics added]. (p. 36)

That the infant cerebellum encodes such body/environment predictive internal models as Vandervert [4, 5] proposed is supported by the following recent research. First, visual-spatial working memory begins to be established by 6 months of age [45]. Second, the growth of neural networks for working memory in the infant are the same as those in older children and adults in connecting frontal, parietal and temporal regions of the brain [46]. Moreover, Knickmeyer, Gouttard, Kang, Evans, Wilber, Smith et al. [47] argued that the 240% increase in the size of the cerebellum in the first year suggested the following:

Because the cerebellum is critically involved in motor coordination and balance [48] the striking cerebellar growth may underpin the rapid motor developments of infancy. The cerebellum has also been implicated in a plethora of other cognitive abilities including planning, set-shifting, language abilities, abstract reasoning, *working memory* [italics added], and *visual-spatial organization* [italics added] [49]. Given that “cognitive” regions of the cerebellum have reciprocal projections with nonprimary frontal, parietal, and occipital association cortex [50], the extremely rapid growth of the cerebellum in the first year may be a prerequisite for specific aspects of later cortical development. ([47], p. 12180)

To support this “prerequisites-for-later-cortical-development” argument it is suggested that the *transition* from visual-spatial working memory toward unconscious inner speech in early developing verbal working memory draws upon the same regions that support motor preparation and planning but not overt motor execution as found for unconscious inner speech in adults by Marvel and Desmond [15], namely, the premotor cortex, pre-SMA and superior cerebellum (Lobule VI and Crus I). This contention is strongly supported by Liao, Kronemer, Yau, Desmond and Marvel [22] who subsequently found that, indeed, nonverbal (pictorial) information draws upon these same motor regions. Mandler’s idea that later, consciously accessible language concepts are built from the infant’s visual-spatial conceptual primitives (Fig. 2) therefore squares well with Knickmeyer, Gouttard, Kang, Evans, Wilber, Smith et al.’s [47] suggestion that the unparalleled growth of the cerebellum in infancy is a prerequisite for the later cognitive development of specific regions of the cerebral cortex.

Vandervert [4, 30] proposed that the *sequence-detection process* described by Akshoomoff, Courchesne and Townsend [8] and Leggio and Molinari [9] (above) is, in fact, the mechanism of the distillation process described by Mandler as noted above. That is, cerebellar sequence detection produces the neural patterning that constitutes the physical world of the infant that we, through additional later-developed internal models, come to know as the “laws” of physics.

Again, it is critically important to note here that Mandler’s conceptual primitives are proposed to be the foundations of symbol systems:

Instead of merely “looking,” the infant notices some aspect of the stimulus array, and recodes it into a simplified form that loses the details of what is being observed, but *distils* [italics added] its meaning. [Vandervert [4, 30, 51] proposed that this occurs in the cerebellum.] The format of the representations that perceptual analysis produces is not propositional; rather, the theory proposes that the earliest meanings appear in the form of analogical representations called image-schemas [alternatively, Mandler refers to these as conceptual primitives]. These early representations are part of the symbolic function in the sense that they are meanings which symbols (gestures, images, or words [or numbers, it is proposed]) refer to or evoke. ([40], p. 277)

It is important to note that Mandler [39] further proposed that movement dynamics (which she referred to as “animacy”) were enfolded via the infant’s scanning into the image-schemas depicted in Fig. 2. Vandervert [38] proposed that each movement and thought is brought toward optimization and the prediction of future events a la Akshoomoff, Courchesne and Townsend’s [8] cerebellar sequence detection.

Based upon the foregoing cerebellar learning and optimization of patterning in the conceptual primitives (the bases for symbol system), it will now be argued that mathematics and physical laws are products of the computational mechanisms of the human cerebellum, and that physical laws are, indeed, the result of the cerebellum’s distillation of the features objects moving in space. Figure 2 illustrates the conceptual primitives first learned in infancy and, collectively, constitute a foundational, rudimentary physics (Vandervert, [4, 30]).

### The beginning of the patterning that becomes mathematics and number in the Cerebro-Cerebellar system (a second tier of cerebellar abstraction)

Through cerebellar distillation the conceptual primitives shown in Fig. 2 are of course *abstractions* based on many repeated perceptual/movement/tactile scans by the infant of its environment. That is, the primitives in Fig. 2 don’t apply/represent particular situations or events but rather apply to all such situations and events in general. However, this is only the beginning point of abstraction or, as Mandler [39] put it, “distillation.” That is, it is a *first tier* of abstraction from which, it is proposed, “number” (the cerebellar basis of mathematics) is concomitantly derived as a second tier of abstraction that is, through the same above repetitions, mapped onto the first tier. This idea is very strongly supported by research that indicates that infants discriminate differing numbers of objects [52–54].

It is further proposed that “number sense” (fluidity or automaticity in number use and understanding) is a product of the fluidity of the manipulation of cerebellar dynamics and inverse dynamics internal models related to this second tier of abstraction or distillation. This idea is strongly supported by Hayter, Langdon and Ramnani’s [34] findings that number manipulation is automated in the cerebellum. We will return to the concept of number sense as a unique product of cerebellar *inverse dynamics models* in the next section.

### The adaptive value of the second tier (number) of Cerebellar abstraction

The explanation of the adaptive value of this second tier of cerebellar abstraction is as follows. It is proposed that to efficiently predict complex future states within the framework of Leggio and Molinari’s ([9]) earlier-quoted sequence detection process (and thereby provide selective advantage in the struggle for survival) the cerebellum must not only learn distilled models of the objects (entities, paths, containers) and dynamics seen in Fig. 2 but must also distil models of the differing *numbers* of entities or persons (or predators) and the dynamics of environments (paths, containers, collectively escape routes) and their relevant *dimensions* (size, length, etc.). That is, it is suggested, within the framework of the apt title of Leggio and Molinari’s article on cerebellar sequence detection, namely, “Cerebellar Sequencing: a trick for predicting the future,” numbers of entities and their dynamics are as important to predicting complex future circumstances among numbers of objects (and thereby survival) as are the objects themselves. Again, this idea is very strongly supported by research that indicates that infants discriminate differing numbers of objects [52–54]. It is worth noting here in regard to Dehaene’s [10] number sense in lower animals that, starting with animal play, animal cerebella would learn these same second-tier object differentiations for the same adaptive reasons as would human infants. For the involvement of the cerebellum in early animal learning, see Vandervert [55].

### The Cerebellar mechanism that differentiates the concept of “number” from collections of objects

#### The Cerebellar origin of “number” in the abstract

But how is “number” in the abstract sense differentiated from collections of objects and their dimensions? Ito [19–21] convincingly argued that when movements and thoughts are repeated (this would of course include the infant’s repetitious observation and interaction with objects moving in space) they are learned as cerebellar *dynamics and inverse dynamics* models, and that these dynamics models learn, not only the individual trajectory actually practiced but generalize for application to quite different (and faster) trajectories of movement and thought.^1^ Accordingly, it is proposed that when cerebellar dynamics models of numbers of objects and motions are learned, they likewise *generalize* (as do movement trajectories) not to just numbers of animals, objects, and motions, etc. but to numbers of “anything.” That is, through the mechanism of cerebellar dynamics modeling of both movements and thoughts about objects and motions, when fed forward to the frontal and parietolateral association areas of the cerebral cortex (Ito, [19]), the concept of “number” becomes an unconscious/potentially-conscious entity in itself. Thereby, numbers can then be applied to imaginary circumstances involving object moving through space. Through accumulated cerebellar optimization of such imagined circumstances, for example, the often imagined circumstances which eventually led to Einstein’s special theory of relativity (see Vandervert [4] for detailed account of Ito [19] applied to Einstein’s own descriptions of his intuitive discovery), new mathematics may constantly emerge. It is suggested that this world of cerebellar dynamics and inverse dynamics models when sent below the level of conscious awareness to the cerebral cortex, constitutes the aspects of the human “imagination” which, at the beginning of this article, Abbott theorized was the source of mathematics.

### The Cerebellar origin of “number sense” in the child

How is the cerebellar modelling of “number sense” differentiated from cerebellar modelling based on the conceptual primitives illustrated in Fig. 2 that lead to language development? Ito [19] further points out that cerebellar dynamics models versus cerebellar *inverse* dynamics models assist the cerebral cortex differently and are controlled by different parts of the cerebellum:

A dynamics model built into the paravermis-interpositus division of the cerebellum enables the motor cortex [or other areas of the cerebral cortex] to direct limb movement [or nonmotor functions] without peripheral feedback. By contrast, an inverse dynamics model built into the hemisphere-dentatus division of the cerebellum replaces the controller task of the motor cortex [or nonmotor areas of the cerebral cortex], rendering the control more automatic and less conscious. Hence, after repeated exercise, one becomes able to move [or think or calculate] quickly, precisely and smoothly without conscious thought. (p. 449)

It should be noted that the entire point of Ito’s above article was to argue extension of the learning of cerebellar dynamics and inverse dynamics models to mental functions. For more detail, the reader is encouraged to consult also endnote-1.

### Fluidity in number sense

Thus, it is suggested that the development of *fluidity* in number sense is more reliant on *inverse dynamics* models (because they result in more automatic or intuitive performance), and language is necessarily more reliant on dynamics models (because it requires constant updating throughout the course of thought or social exchange). It is hypothesized that since the learning of *inverse dynamics* models first requires the learning of *dynamics* models [19] and then further requires continued levels of practice (specifically with number tasks in the case of the development of number sense), inverse dynamics models are more difficult to learn for some children depending on, for example, the child’s history of exposure to learning number operations or his/her executive capacity in working memory to continue their focus attention on repetitive tasks. The role of the executive component of working memory in such learning will be discussed below.

The cerebellar modeling of the additional features of number, dimensions, and motion related to processes of imagination applies in common across all of the primitives and their mutual relationships seen in Fig. 2. *This secondary, more abstract distillation, it is proposed, becomes the basis of mathematics as we come to know it and develop it through additional scientific and technological models, its constant elaborations.* Accordingly, during both the advancement of societies and the enculturation of each individual (Vandervert, [5]) this second tier of largely cerebellar inverse dynamics modeling (mathematics) is constantly unconsciously elaborated in cerebellar inverse dynamics models of each individual as generations of children learn accumulations of science, technology, and so forth in school, occupations and professions of society. These advanced cerebellar models are examples of what Leggio and Molinari [9] were in part referring to in their cerebellar Sequence Detection Hypothesis as, “inferences of higher-order processes (e.g., cognitive elaboration or social cognition),” (p. 2). It is further suggested that in prehistory, this foundational basis of mathematics in the cerebellum only became *conscious* rudimentary “mathematics” when blended (from multiple cerebellar internal models [32]) with other cultural requirements for, for example, learning the sequence of steps in the making of composite tools, in the enumeration related to the stringing of shells which might have culturally shared significance, or, in children, the demands of enculturation, including of course in modern times, schooling [38].

### Implications for developmental dyscalculia

Although the main arguments of this article are aimed at understanding the ultimate foundations of mathematics in internal models of the cerebellum, the implications of the foregoing proposal that the basis of number and mathematics lies in a second tier of abstraction in the cerebellum naturally has important implications for understanding *developmental dyscalculia*. Dyscalculia refers to a difficulty in learning or comprehending, arithmetic such as difficulty in understanding numbers, learning how to manipulate numbers, and learning arithmetic facts. It is generally understood as a developmental disorder. For additional details on the nature of dyscalculia see Kaufmann and von Aster [56], Landerl, Bevan and Butterworth [57] and von Aster and Shalev [58], and for models of developmental dyscalculia that are compatible with Vandervert’s [4, 5, 30] interpretation of the cerebellar basis of Mandler’s (Fig. 2) conceptual primitives that includes working memory, executive control, and visual-spatial learning, see Verdine, Golinkoff, Hirsh-Pasek and Newcombe [59] and von Aster and Shalev [58].

It has been shown that fluidity of movement and thought (speed, consistency, and appropriateness) is governed by predictive sequence detection in the cerebellum [8, 9, 12, 13, 19–21]. It is suggested that difficulty with “number sense” (again, defined as fluidity or automaticity in number use and understanding) may originate in the developmental transition from visual-spatial working memory in infancy (see Fig. 2 and accompanying discussion) toward executive control related not to accessibility to the first tier of conceptual primitives for objects and movements, but to the second tier of abstraction toward “numbers” via *verbal* working memory. The proposed difference in difficulty in the early developmental learning of these two tiers of abstraction is discussed above in relation to the extended learning requirements of *inverse* dynamics models. This more demanding second-tier access explanation might explain why those with developmental dyscalculia may have difficulty with number sense, but not with language. That is, the problem of dyscalculia may be the result of problems with the development of only the second tier of abstraction in mostly cerebellar inverse dynamics models which differentiate “number” from collections of objects, their movements, and their dimensions.

### Number and mathematics (and counting) begin in the Cerebro-Cerebellar system, not in the cerebral cortex alone

There is a rather large and lengthy tradition of research on the use of the fingers in *improving* (not originating) counting and calculation in arithmetic (Andres, Michaux & Pesenti, [60]; Kaufmann, [61]; Nöel, [62]). Researchers in this traditional approach have generally focused attention on finger counting and “number sense” exclusively in the cerebral cortex (Kaufmann, [61]).^2^ Specifically, attention has been focused especially on the parietal regions and, notably, the intraparietal sulcus (Andres, Michaux & Pesenti, [60]).

As stated earlier in the purposes section, the cerebro-cerebellar approach of this article does *not* necessarily conflict with mathematics or number sense models that focus on brain functions associated with especially the intraparietal sulcus region of the cerebral cortex, for example, Dehaene [10], Dehaene, Spelke, Stanescu, Pinel and Tsivkin [11]. The cerebral cortex is of course involved in the here-and-now conscious manipulation of number and mathematics. However, in this regard, Vandervert [4, 5] has pointed out that like the long histories of the cultural developments of technology, science, engineering and music, mathematics and number sense too have only been possible as products of repetitious practice within the individual or within generation-after-generation cerebro-cerebellar advances that, in their *origins*, have been prominently driven by the predictive sequence-detecting computations of the cerebellum.

### Pattern detection and optimization: The neural “machine” behind the intuitive aspects of mathematics and number sense

As described earlier, it is widely understood that such *practiced* improvements in number calculation (or in anything else practiced) would be learned as constantly error-corrected internal models in the cerebellum (Akshoomoff, Courchesne & Townsend, [8]; Ito, [20, 29]; Leggio & Molinari, [9]; Leiner, Leiner & Dow, [12, 13]). And, it is proposed that, because it sets the occasion for structured practice, the use of fingers in calculation improves arithmetic ability via the cerebellum-driven collaboration between the cerebellum and cerebral cortex as described in some detail in Akshoomoff, Courchesne and Townsend’s [9] following cerebellar sequence-detection process of prediction:

The cerebellum is a master computational system that adjusts responsiveness in a variety of networks to obtain a prescribed goal. These networks include those thought to be involved in declarative memory, working memory, attention, arousal, affect, language, speech, homeostasis, and sensory modulation as well as motor control…We hypothesized that the cerebellum does this by *encoding (“learning”) temporally ordered sequences* of *multi-dimensional information* [italics added] about external and internal events (effector, sensory, affective, mental, autonomic), and, as similar sequences of external and internal events unfold, they elicit a readout of the full sequence in advance of the real-time events. This readout is sent to and alters, *in advance* [italics added], the state of each motor, sensory, autonomic, attentional, memory, or affective system which, according to the previous “learning” of this sequence, will soon be actively involved in the current real-time events. So, in contrast to conscious, longer time-scale anticipatory processes mediated by cerebral systems, output of the cerebellum provides moment-to-moment, *unconscious* [italics added], very short time-scale, anticipatory information. (p. 592)

Akshoomoff, Courchesne and Townsend’s foregoing account of cerebellar sequence detection is in agreement with Leggio and Molinari’s [9] sequence-detection process described earlier, but is presented separately here because it clearly sets out additional aspects of the overall process. For example, Akshoomoff, Courchesne and Townsend (1) spell-out the cerebellar involvement of “*temporally ordered sequences* of *multi-dimensional information”* (working memory, attention, language and motor manipulations), and (2) make the point that this cerebellar sequence detection takes place below the level of conscious awareness (unconsciously). We will return to these aspects of cerebellar sequence detection in a moment.

### What exactly happens in working memory during the development of number sense, and how might it be related to dyscalculia?

How, exactly, would the cerebellum be involved in the development of number sense and/or the working memory of finger calculation? Marvel and Desmond [15–17] have shown that silent inner speech (speech which may not reach consciousness) in the cerebellum indicates central executive control of verbal working memory manipulation of tasks. In such working memory tasks, they found the executive control in cerebellar inner speech to be associated with *motor planning and preparation related to encoding and retrieval* of task information (Marvel & Desmond, [15]). Specifically, they found that encoding information into working memory increased dorsal dentate (motor) activity in the cerebellum, while in the retrieval phase of working memory activity increased in the cerebellar ventral dentate (cognitive). In their conclusion, they proposed that:

The cerebellum enhances working memory by supporting inner speech mechanisms. This capability emerged from overt speech and motor systems as an evolutionarily adaptive way to *boost cognitive processes* [italics added] that rely on working memory, such as language acquisition. ([15] p. 7)

In a review of the cerebellum and nonmotor functions, Strick, Dum and Fiez [25] strongly supported this facilitative (and elaborative) role of cerebellar inner speech in working memory. They suggested that the cerebellum is recruited whenever people engage in inner speech “to represent, maintain and organize task-relevant information and conscious thoughts” (p. 426), including in, for example, verbal working memory.

### How Cerebellar inner speech accesses number sense

But how does unconscious inner speech in the cerebellum *boost* cognitive processes in verbal working memory, as Marvel and Desmond [15] proposed? Moreover, what is the underlying *cerebellar mechanism* that drives its unconscious inner speech, “to represent, maintain and organize task-relevant information and conscious thoughts” (Strict, Dum & Fiez, ([25], p. 426))? What was/is its evolutionary *adaptive* mechanism?

To answer this question, it is proposed that what Marvel and Desmond [9] refer to above as cerebellar *motor planning and preparation* is evolutionarily adaptive in boosting cognitive processes, because, as in all skill development, cerebellar inner speech is driven by Akshoomoff, Courchesne and Townsend’s [8] and Leggio and Molinari’s [9] cerebellar sequence detection process as described earlier in this article. That is, Marvel and Desmond’s motor and *planning and preparation* is in actuality a succinct and equivalent way of describing Akshoomoff et al.’s and Leggio and Molinari’s *prediction and anticipatory system adjustments*. The precise planning (anticipatory) mechanism that is evolutionarily adaptive can be readily appreciated by paraphrasing Akshoomoff et al.’s [8] sequence detection scenario within the context of Marvel and Desmond’s above-cited conclusions on the role of cerebellar inner speech as follows:

Inner speech in the cerebellum boosts executive processes in working memory by *encoding (“learning”) temporally ordered sequences* of multi-dimensional information about external and internal events *(effector, sensory, affective, mental, autonomic)*[italics added], and, as similar sequences of external and internal events unfold, they elicit a readout of the full sequence in advance of the real-time events. This readout is sent to and alters, *in advance* [italics added], the state of each motor, sensory, autonomic, attentional, memory, or affective system which, according to the previous “learning” of this sequence, will soon be actively involved in the current real-time events. *So, in contrast to conscious, longer time-scale anticipatory processes mediated by cerebral systems, output of the cerebellum provides moment-to-moment, unconscious [italics added], very short time-scale, anticipatory information* [italics added]*.* (Paraphrased by combining Akshoomoff et al. ([8], p. 592) with Marvel & Desmond [15]

Evidence that Boosts in Fluency in Number Sense Occur in Inner Speech of Verbal Working Memory in the Cerebellum

As describer earlier in this article, Hayter, Langdon and Ramnani [34] conducted imaging research on the involvement of the cerebellum in arithmetic calculation in verbal working memory. Recall that Hayter et al. used the Paced Auditory Serial Addition Test (PASAT) where subjects sequentially added the last two numbers in an overall sequence of five numbers. During this number addition task in verbal working memory they found that Crus VII in the cerebellar cortex along with prefrontal and parietal areas of the cerebral cortex were involved in automatic counting. However, this cerebellar role in automated counting or number sense (via dynamics and inverse dynamics internal models) had not been related to its roles in number sense or dyscalculia.

### Possible location of number sense in internal models in the cerebellum

When placed within the context of dyscalculia, an interesting quirk of methodological circumstance can be seen in Marvel and Desmond’s [15, 16] research on inner speech in the cerebellum. While counting was not the point, per se, of Marvel and Desmond’s research method, the task their working memory “executive condition” subjects completed was, besides being a cognitive task, was coincidentally a *counting* task. Specifically, in their retrieval phase, retrieval in the executive working memory condition required subjects to count two letters forward in the alphabet from their original encoded target letter in order to complete the task. For the typical subject this minimal counting is an innocuous task. However, for the dyscalculia subject this requirement of quickly counting ahead two letters in the alphabet would clearly present a significant additional attentional control (executive) demand on working memory over that experienced by the typical subject. That is, for subjects with dyscalculia this counting requirement during retrieval would put considerable additional load on the executive retrieval phase of working memory. As mentioned earlier, models of developmental dyscalculia are compatible with Vandervert’s [4, 5, 30] interpretation of the cerebellar basis of Mandler’s (Fig. 2) conceptual primitives that include working memory, executive control, and visual-spatial learning, see Verdine, Golinkoff, Hirsh-Pasek and Newcombe [59] and von Aster and Shalev [58]. However, these models do not include studies of the possible contributions of inner speech in the cerebellum.

### Cerebellar inner speech in a hypothetical dyscalculia subject

For the typical subject in Marvel and Desmond’s [15, 16] research methodology the retrieval (cognitive) task resulted in *more* activity in the cerebellar ventral dentate. In a hypothetical dyscalculia subject on the other hand, it is proposed that, because this task involved counting, there would be *less* activity in the ventral dentate during the retrieval phase. Less activity in the ventral dentate would be interpreted to indicate that the ventral dentate in the dyscalculia subject is deficit (perhaps due to genetic or health factors) in learning inverse dynamics models behind number sense. While such a study of this proposed relationship between cerebellar inner speech and dyscalculia is beyond the scope of the present article, it has important theoretical implications for understanding a more precise locus of the development of dyscalculia that includes the refinements of skill that are unique to the contributions to number sense via inner speech in the cerebellum.

### Fingers and inner speech applied together during counting may be seen as a dorsal-to-ventral dentate “marriage” in the cerebellum

**Fig. 3:**
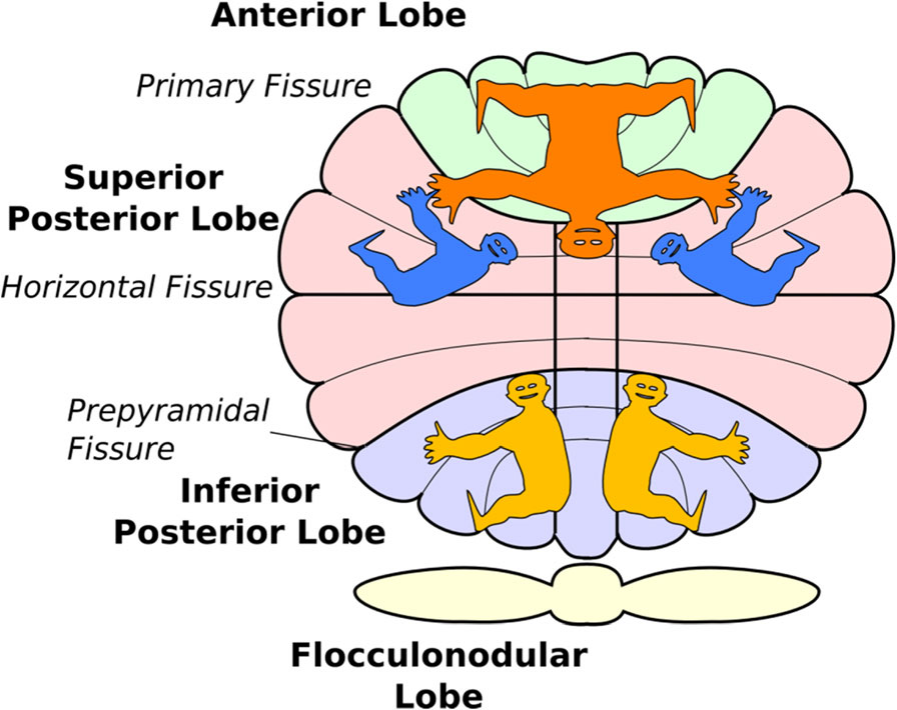
Somatotopic Maps of Cerebellum: Fingers in the most crucially relevant sensory/motor homunculi for this article appear in lobule VI (*upper pink*). Lobule VI is linked to the frontal and parietal areas of the cerebral cortex. Multiple body representations within the cerebellum: Shown are the anterior (*top, orange*) and inferior posterior (*bottom, yellow*) body representations, as well as the newly identified superior posterior representation (*blue*). (Adapted from Snider & Eldred, [73]; Grodd et al., [74]; Schlerf et al., [63]). Reprinted with permission from John Schlerf as the figure appears in: John Schlerf, Tobias Wiestler, Timothy Verstynen and Joern Diedrichsen [64]

As cited earlier, counting and calculation with the fingers improves skills in arithmetic (Kaufmann, [61]). The following hypothesis is proposed to explain this relationship. Counting and calculation in the executive inner speech of verbal working memory in the cerebellum, is paired with parallel sequencing of executive attentional control of the finger motor areas of the cerebellum. Note the finger portions in the somatotopic maps of the cerebellum in Fig. 3.

In support of this hypothesis Schlerf, Verstynen, Ivry and Spencer [63, 64], from which the somatotopic map in Fig. 3 was adapted, concluded as follows:

Complex finger movements produce activation along the medial surface of the left precentral gyrus, and this activation is similar for both left and right hand movements (Hanakawa et al. [65]; Verstynen et al. [66]). The anatomical, premotor location of this activity, coupled with the bilateral pattern of activation, favors a motor planning account. By analogy, we propose that the neocerebellar activity also reflects high level motor planning rather than the processing of sensory signals. While this hypothesis is admittedly speculative, the main point to be emphasized here is that the activation is somatotopic, arguing against functional hypotheses that are divorced from the sensorimotor domain. ([63], p. 3335)

Within the context of the foregoing motor-planning account, it should be further recognized that the finger representations in the cerebellum (Fig. 3) provide skeletomuscular models of the fingers in the form of Ito’s [20] cerebellar corticonuclear microcomplexes (CNMC’s). (Cerebellar microcomplexes are the error-driven, adaptive neural basis of cerebellar dynamics and inverse dynamics internal models [21].) Since these error-driven cerebellar microcomplexes of *potential* finger/hand manipulation are prewired from birth to finger/hand areas in the motor-sensory areas of the cerebral cortex, it is suggested they can be quite readily (1) blended (a la Imamizu, Higuchi, Toda and Kawato [32]) (2) in accordance with cerebellar sequence prediction described by Leggio and Molinari [9] with CNMC’s associated with silent cerebellar inner speech described by Marvel and Desmond [15]. It is suggested that this would, thereby, enhance executive control of speech in working memory as proposed by Marvel and Desmond [15–17]. That is, in this way, finger counting would positively enhance the development of central executive control in arithmetic tasks as found by Andres, Michaux and Pesenti, [60]; Kaufmann, [61]; and Nöel, [62].

Thus, it can be argued that, in addition to extending executive control toward the learning of cerebellar inverse dynamics models, counting with the fingers *improves* (does not originate) arithmetic skills, because it combines the two parallel, mutually reinforcing lines of sequence detection during the learning of (1) cerebellar motor planning of finger representations in the cerebellum and (2) motor traces of inner speech in verbal working memory. It should be noted that this would combine the dorsal and ventral dentate connections with cerebellar lobules VI and VII with loops to/from frontal and parietal areas of the cerebral cortex (Leiner, Leiner & Dow, [12]; Stoodley, Valera & Schmahmann, [24]).

There is no reason not to believe that these cerebellar motor traces also occur (arise in relation to) in the evolutionarily older somatotopic maps in the posterior lobes of the paleocerebellum (bottom, blue area in Fig. 3). It is perhaps largely through these older somatotopic mappings that the preverbal infant interacted repetitively with its environment of produce (distil) the foundational physics shown in Fig. 2. This idea comports nicely with (1) Gogtay, Giedd, Lusk et al.’s [67] findings that evolutionarily older brain regions mature *earlier* than phylogenetically younger regions, and (2) with Marvel and Desmond’s [15] following insightful elaboration of Gogtay et al.’s critically important point: “It therefore seems plausible that throughout development the cognitive loop [ventral dentate] retains close ties with its evolutionary precursor, the motor loop [dorsal dentate], which allows both systems to work together, for example by engaging inner speech mechanisms to enhance working memory,” ([67] p. 274). In light of the multiple somatotopic mappings shown in Fig. 3, it is suggested that in this dorsal-to-ventral dentate manner, the executive use of fingers in counting produces dual, perhaps triple, motor traces in parallel with executive cerebellar inner speech to enhance executive control in working memory. This dorsal-to-dentate scenario would thereby tend to improve (again, not originate) the execution of arithmetic skills.

## Discussion

It is suggested that the computation of patterning in internal models in the cerebellum is predominantly responsible for creating our physical realities, laws, and numbers and offers a neuroscience justification for Derek Abbott’s [3] view that mathematics originates in human thought. That is, the cerebellum is the predominant source of patterning that becomes mathematics, the science of patterns [6]. Evidence that the ultimate origin of mathematics and number is principally cerebellum-driven in the cerebro-cerebellar system is based on the 69-billion-neuron computational power of cerebellar internal models, operating below the level of conscious awareness, ultimately produces our models of physical reality, number and mathematics.

### The Cerebro-Cerebellar approach does not conflict with traditional brain models of mathematics and number sense

Importantly, it is pointed out that the cerebro-cerebellar approach does *not* necessarily conflict with mathematics or number sense models that focus on brain functions associated with especially the intraparietal sulcus region of the cerebral cortex, for example, Dehaene [10], Dehaene, Spelke, Stanescu, Pinel and Tsivkin [11]. Rather, the cerebro-cerebellar approach brings to bear additional brain mechanisms that may provide more detailed and more comprehensive explanations for (1) the initial learning of number and its manipulation, and (2) the subsequent, ongoing optimization and increased complexity of the neural patterns that constitute both mathematics and number sense. In this regard, two explanatory advantages are immediately evident: (1) the “intuitive” character of number sense (Dehaene [10]) can be parsimoniously and definitively explained in terms of unconsciously learned internal models in the cerebellum, which are then sent to the cerebral cortex, and (2) number sense in lower animals (Dehaene [10]) can likewise be parsimoniously and definitively explained in terms of unconscious internal models learned in animal cerebella, which are then sent to their respectively developed cerebral cortices.

Within this cerebro-cerebellar framework it is suggested that (1) during infancy the cerebellum learns a first tier of internal models based on the infant’s perception and movement which results in a primitive physics that constitutes the foundations of visual-spatial working memory, (2) at the same time a second (and more abstract) tier of cerebellar *inverse* dynamics models based on the first tier (physics) learns number and relationships among dimensions across those primitive physics of the first tier, and (3) “number sense” originates largely below the level of consciousness in the cerebellar *inverse* dynamics models described in (2). *This general view is strongly supported by findings that the cerebellum automates the manipulation of number information* [34]*.* Further, developing from this conceptual structure of internal models, the cerebellum’s developing inner speech both boosts verbal working memory capacity and enhances executive control processes in that working memory and silent speech-enhanced executive control in verbal working memory [15–17] facilitates the learning of inverse dynamics models related to the development of number sense.

The foregoing framework for the ultimate origin of number and mathematics involves cerebro-cerebellar loops between the cerebellum on the one hand and the frontal and parietal regions of the cerebral cortex on the other [17]), however (and this is critically important), the ultimate origin and creative expansion of number and mathematics is driven principally by the predictive, error-corrective *sequence detection mechanism* in the cerebellum (Akshoomoff, Courchesne & Townsend, [8]; Leggio & Molinari, [9]). This prominent role of the cerebellum is secondarily supported by cerebro-cerebellar loops connecting at least two somatotopic finger representations in the cerebellum (Fig. 3) and inner speech-driven finger counting/calculation which produces parallel, mutually strengthening and sequentially structured motor traces to *boost* (not originate) the manipulation of numbers in verbal working memory. Since it is widely accepted that improvement in any motor or cognitive skill is accomplished through the *repetitive* learning (practice) that constitutes cerebellar internal models (Doya, [31]; Ito, [19, 29]; Leiner, Leiner & Dow, [12]), it is suggested that it is the structured cerebellar modeling related to Fig. 3 along with its related cerebellar inner speech that is the basis for the findings that finger counting improves arithmetic ability (Andres, Michaux & Pesenti, [60]; Kaufmann, [61], Nöel, [62]). That is, it is proposed that the practice-driven, constantly error-corrected, anticipatory information related to finger counting improves the “orchestration” of the central executive, just as does the timing and sequencing in playing a musical instruments (Vandervert [4]).

## Conclusion

It is concluded that (1) the cerebellum is the predominant source of patterning and optimization that produces mathematics in the cerebral cortex, and (2) difficulty with “number sense” in dyscalculia originates in the developmental transition from visual-spatial working memory in infancy (see Fig. 2 and accompanying discussion) toward inner speech associated with executive control related to accessibility to the second tier of abstraction. It is proposed that this may occur during the retrieval phase of verbal working memory which has been found to be a function of the ventral dentate of the cerebellum [15, 16]. *It is further proposed that this second tier learning consists mostly of inverse dynamics models which require extended focus of attention in repetitious tasks.* This two-tier explanation might explain why those with developmental dyscalculia may have difficulty with number sense, but not with language which is more based on the conscious manipulation of dynamics models as opposed to *inverse* dynamics models. That is, the problem of dyscalculia may be the result of problems with the transitional development of only the second tier of abstraction which differentiates “number” from collections of objects and their dimensions. In this regard, Hayter, Langdon and Ramnani [34] established that verbal working memory in the cerebellum is involved in the automaticity of number manipulation. And, as pointed out earlier, Marvel and Desmond [15, 16] have operationalized components of verbal working memory (encoding, maintenance, and retrieval) toward number manipulation in the cerebellum, it is suggested that, while beyond the scope of the purposes of this article, the latter’s methodology can be modified to study this transition in relation to the development of number sense, for example, in those who later develop difficulty with numbers, including dyscalculia.

## Footnotes

1 A detailed explanation of the theoretical basis and description of dynamics and inverse dynamics cerebellar internal models is beyond the scope of this article. The interested reader may consult Ito [19, 20] for easy to understand descriptions of these terms related to both movement and thought. For a more rigorous theoretical/mathematical account, including definitions of dynamics versus inverse dynamics cerebellar models see Kawato, Furukawa and Suzuki [68]. For application of cerebellar dynamics and inverse dynamics models to higher-order thought processes see Imamizu and Kawato [69].

2 It is noted here that Arsalidou and Taylor [70] found cerebellar activity during calculation tasks (addition, subtraction, multiplication), however no analysis was provided that described the role of this cerebellar activity in that calculation. Moreover, in following work on mathematical functions in the brain (Klein, Moeller & Willmes [71]; Klein, Suchan, Moeller, et al. [72]) that was both in direct line with and cited Arsalidou and Taylor’s general findings and conclusions, there was no further mention of involvement of the cerebellum.
